# Supplementary material for: Mechanisms governing the pioneering and redistribution capabilities of the non-classical pioneer PU.1
Source: Nat Commun. 2020 Jan 21;11:402. doi: 10.1038/s41467-019-13960-2 (PMC6972792; doi:10.1038/s41467-019-13960-2)
Supplement: Supplementary file 7 — Source data [file 41467_2019_13960_MOESM7_ESM.zip › Source_Data/FigureS2/FigureS2B_MotifScanOutput/KmeansCluster3/homerResults/motif46.similar.html]

motif46

## Information for motif46

G
T
C
A
G
T
A
C
G
T
C
A
A
C
G
T
T
A
G
C
G
A
T
C
G
A
C
T
G
T
A
C
G
C
A
T
C
A
G
T
A
G
C
T
A
G
C
T
  
Reverse Opposite:  

T
C
G
A
T
C
G
A
G
T
C
A
C
G
T
A
C
A
T
G
C
T
G
A
C
T
A
G
A
T
C
G
T
G
C
A
C
A
G
T
C
A
T
G
C
A
G
T
  

|  |  |
| --- | --- |
| p-value: | 1e-21 |
| log p-value: | -5.005e+01 |
| Information Content per bp: | 1.578 |
| Number of Target Sequences with motif | 42.0 |
| Percentage of Target Sequences with motif | 0.88% |
| Number of Background Sequences with motif | 34.8 |
| Percentage of Background Sequences with motif | 0.08% |
| Average Position of motif in Targets | 106.2 +/- 46.3bp |
| Average Position of motif in Background | 111.3 +/- 63.2bp |
| Strand Bias (log2 ratio + to - strand density) | -0.1 |
| Multiplicity (# of sites on avg that occur together) | 1.02 |
| Motif File: | file (matrix) reverse opposite |

### Similar de novo motifs found

|  |  |  |  |  |  |  |  |
| --- | --- | --- | --- | --- | --- | --- | --- |
| Rank | Match Score | Redundant Motif | P-value | log P-value | % of Targets | % of Background | Motif file |
| 1 | 0.927 | A G T C G T C A G T A C C G T A A C G T A G T C A G T C A G C T G T A C C G A T C A G T A G C T A G C T | 1e-19 | -44.797412 | 0.63% | 0.03% | motif file (matrix) |
